# Serum neprilysin levels are associated with myocardial stunning after ST-elevation myocardial infarction

**DOI:** 10.1186/s12872-020-01578-y

**Published:** 2020-07-02

**Authors:** Damien Legallois, Clémence Macquaire, Amir Hodzic, Stéphane Allouche, Ismaïl El Khouakhi, Alain Manrique, Paul Milliez, Eric Saloux, Farzin Beygui

**Affiliations:** 1 Department of Cardiology, EA4650 Signalisation, Electrophysiologie et imagerie des lésions d’ischémie-reperfusion myocardique (SEILIRM), FHU REMOD-VHF, Normandie Univ, UNICAEN, CHU de Caen Normandie, 14000 Caen, France; 2 Department of Cardiology, Normandie Univ, UNICAEN, CHU de Caen Normandie, 14000 Caen, France; 3 Department of Clinical Physiology, INSERM Comete, Normandie Univ, UNICAEN, CHU de Caen Normandie, 14000 Caen, France; 4 Department of Biochemistry, EA4650 Signalisation, Electrophysiologie et imagerie des lésions d’ischémie-reperfusion myocardique (SEILIRM), FHU REMOD-VHF, Normandie Univ, UNICAEN, CHU de Caen Normandie, 14000 Caen, France; 5 Department of Nuclear Medicine, EA4650 Signalisation, Electrophysiologie et imagerie des lésions d’ischémie-reperfusion myocardique (SEILIRM), GIP Cyceron, FHU REMOD-VHF, Normandie Univ, UNICAEN, CHU de Caen Normandie, 14000 Caen, France; 6 ACTION academic research group, Pitié Salpêtrière University Hospital, Paris, France

**Keywords:** Neprilysin, ST-elevation myocardial infarction, Left ventricular remodeling

## Abstract

**Background:**

Left ventricular remodeling following ST-elevation myocardial infarction (STEMI) is associated with poor outcome, including heart failure (HF). Neprilysin inhibition leads to improved outcome in patients with altered left ventricular ejection fraction (LVEF).

**Methods:**

We aimed to assess the association between serum levels of neprilysin and left ventricular (LV) volumes, function and remodeling in STEMI patients with successful myocardial reperfusion and no clinical sign of HF. Sixty-eight patients were admitted for STEMI and had both plasma neprilysin measurement at baseline and 3D transthoracic echocardiogram at baseline and after a median follow-up of 7 months. We compared 3 groups: a group with a low-level of plasma neprilysin (< 125 pg/mL, i.e. the lower limit of detection of the assay) and the two other groups were defined as being below or above the median value of the remaining samples.

**Results:**

Median age was 58.5 ± 12.8 years and 56 (82.4%) were men. Median LVEF was 45.0 ± 8.5%. Baseline characteristics were comparable between groups (low-level of neprilysin group [≤125 pg/mL, *n* = 38], medium-level of neprilysin group [126–450 pg/mL, *n* = 15] and a high-level group [> 450 pg/mL, n = 15]). At baseline there was a non-significant trend towards lower end-diastolic volume (*p* = 0.07) but significantly lower LVEF in the high neprilysin group (46.4 ± 8.3%, 47.1 ± 8.1% and 39.1 ± 6.9%, *p* < 0.01). At follow-up, the magnitude of LVEF increase was significantly more important in the high neprilysin group compared to the other groups (*p* = 0.022 for relative change in LVEF and 6.6 ± 7.3%, 3.6 ± 9.0% and 11.3 ± 8.4%, *p* = 0.031 for absolute change in LVEF) resulting in similar LVEF levels at follow-up between all groups (53.0 ± 8.9%, 50.6 ± 9.7% and 50.4 ± 9.9%, *p* = 0.55).

**Conclusions:**

Initial high neprilysin levels may identify patients with stunned myocardium early after STEMI, with a recovery of contractility leading to improved LVEF at follow-up. Future studies will have to assess the role of neprilysin in the setting of STEMI and the potential benefit of its blockade.

## Background

Despite widespread urgent coronary revascularization in the setting of ST-elevation myocardial infarction (STEMI), subsequent left ventricular remodeling (LVR) remains common [1, 2] and associated with mortality, heart failure (HF) and ventricular arrhythmia [3]. LVR is the consequence of cellular and histological modifications, such as myocyte hypertrophy, apoptosis and extracellular matrix remodeling [4]. These phenomena are induced by deleterious adaptive mechanical and neurohormonal responses, including the renin-angiotensin-aldosterone system (RAAS) [5]. Early angiotensin-converting enzyme (ACE) inhibition following STEMI associated with a significant reduction in mortality [6] and decreased LVR through suppression of the activity of the RAAS [7] is to be considered in all STEMI patients [8]. Neprilysin is a neutral endopeptidase that degrades several endogenous vasoactive peptides, such as bradykinin, natriuretic peptides and adrenomedullin [9, 10]. Its inhibition interacts with the RAAS, increasing angiotensin-II blood concentrations as compared with placebo, indicative of blockade of the angiotensin-II receptor type 1 (AT1) receptor [11]. In the PARADIGM-HF trial [12], neprilysin inhibition combined with inhibition of AT1 receptors was superior to ACE inhibition by enalapril in reducing both the risks of death and hospitalization for HF in patients with HF and reduced LVEF. In a recent study, the use of an angiotensin receptor-neprilysin inhibitor yielded a significant decrease in left ventricular (LV) volumes at 4 months in the same HF population [13]. There is no data available about the relationship between baseline neprilysin levels and LVR after STEMI. The aim of the present study was to assess the association between serum levels of neprilysin and LV volumes, function and remodeling in STEMI patients at baseline and 6 ± 1 month follow-up.

## Methods

This study was a prospective, observational multicenter study that included consecutive patients admitted for STEMI and treated with either primary percutaneous coronary intervention (pPCI) or rescue PCI after unsuccessful fibrinolysis therapy, from January 2017 to October 2018. Inclusion criteria were as follows: age ≥ 18 years, chest pain associated with an ECG with ST-segment elevation (either > 1 mm in ≥2 contiguous limb leads or > 2 mm in ≥2 contiguous precordial leads or new left bundle branch block or new significant Q wave). Criteria for exclusion were unsuccessful revascularization (residual stenosis > 30% in the culprit lesion and/or thombolysis in myocardial infarction flow < 3), clinical signs of HF as defined by Killip Kimball class≥II, non-related heart conditions with estimated life expectancy < 12 months and follow-up planned in another center. Informed consent was obtained from the patients. The study complied with the Declaration of Helsinki and was approved by the local ethics committee (protocol number A14-D17-VOL.20).

On admission, routine blood samples were drawn and collected in tubes containing lithium heparin, ethylenediaminetetraacetic acid or spray-coated silica, centrifuged for 12 min at 2000 g at room temperature then assayed for routine biological measurement. The remaining plasma and serum were stored at − 80 °C until use. Neprilysin was measured on remaining plasma samples in duplicate using an enzyme-linked immunosorbent assay (ELISA) kit (Human Neprilysin DuoSet ELISA, RD systems, Minneapolis, USA) according to the manufacturer’s instructions. Data were linearized by plotting the log of neprilysin concentrations versus the log of the optical density and the best fit line was determined by regression analysis. Other measurements specific to the present study included high-sensitivity cardiac troponin I (hs-cTnI) (Dxi Beckman Coulter) and N-terminal pro-B-type natriuretic peptide (NT-proBNP) (Cobas e-411, Roche). Minimum sensitivity and upper limit of detection were 2.3–27027 pg/mL for hs-cTnI, 5–35000 pg/mL for NT-proBNP, and 125–8000 pg/mL for neprilysin; values higher than the upper limit of detection were manually diluted for neprilysin. The highest intra- and inter-assay coefficients of variation were 3.9 and 5.1% for hs-cTnI and 2,7% and 4,6% for NT-proBNP. The recommended diagnostic threshold for the diagnosis of acute myocardial infarction was hs-cTnI > 17.5 pg/mL.

A total of 94 patients with STEMI underwent successful revascularization during the inclusion period. All included subjects had a complete 3D-transthoracic echocardiogram within 48 h. Transthoracic echocardiograms was planned at 6 months after STEMI, as a part of routine follow-up. All echocardiograms were performed using an ultrasonic device system (EPIQ 7G, Phillips Healthcare, Best, Netherlands) and were obtained by experienced ultrasonographers who were unaware of neprilysin measurement results. A standard imaging protocol was used with 4-chamber, 2-chamber apical, and long and short axis parasternal views. Left ventricle end-diastolic volume (EDV), left ventricle end-systolic volume (ESV)) and LVEF were measured with 3D method, using Intellispace Cardiovascular software (Philips Healthcare, Best, Netherlands) and TomTec software (TomTec Imaging Systems GmbH, Unterschlessheim, Germany).

The sample size was estimated based on the assumption of a normal distribution for most variables, either as such or after log-transformation by the inclusion of ≥60 patients. The study was designed to compare 3 groups based on the tertiles of neprilysin concentration in our population. However, because of the skewed distribution of neprilysin values, we decided to define the lowest group by those with levels < 125 pg/mL (i.e. the lower limit of detection of the essay) and to divide the remaining patients into two other groups according to median neprilysin concentration value (450 pg/mL) when quantifiable. Qualitative variables are shown as count and frequency (%). Quantitative variables are presented as mean ± SD or median and interquartile ranges when they had a skewed distribution. Continuous variables were compared using either ANOVA or Kruskall-Wallis test and categorical variables were compared by the χ2 test or Fisher’s exact test, where adapted. Biomarkers levels with skewed distribution were log-transformed before being used as continuous variables in statistical analyses. Spearman correlation coefficient was used to assess the correlation between non-normally distributed quantitative parameters. Statistical tests were 2-sided and used a significance threshold of *p* < 0.05. All statistical analyses were performed using R version 3.4.4 (R Foundation for Statistical Computing, Vienna, Austria).

## Results

**Fig. 1:**
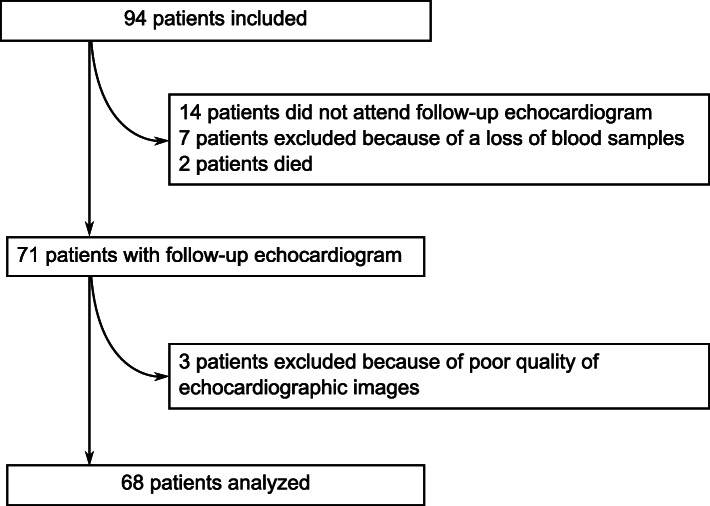
Flow chart

Among the 94 patients with complete 3D-transthoracic echocardiogram at baseline, two patients died during follow-up. Out of the remaining 92 patients, 14 did not attend follow-up echocardiogram, 3 were excluded due to poor quality of echocardiographic images and 7 were retrospectively excluded because of a loss of blood samples (Fig. 1). A total of 68 patients with complete biological data and both initial and follow-up echocardiography was included in the analyis. Baseline characteristics are depicted in Table 1. Median age was 58.5 ± 12.8 years and 56 (82.4%) were men. Baseline LVEF was 45.0 ± 8.5% (Table 2). Drug therapy at baseline and at follow-up is depicted in Table 3. The mean follow-up time was 7 months [6 to 10 months].

**Table 1:** Baseline characteristics according to neprilysin levels

	Overall (*n* = 68)	Neprilysin level ≤ 125 pg/mL (*n* = 38)	Neprilysin level 126–450 pg/mL (*n* = 15)	Neprilysin level > 450 pg/mL (*n* = 15)	Overall *p*.value	Highest-level vs. lower groups *p*.value
Age, years	58.5 ± 12.8	59.8 ± 11.7	54.8 ± 9.8	60.5 ± 16.8	0.37	0.58
Gender, male	56 (82.4%)	31 (81.6%)	13 (86.7%)	12 (80.0%)	0.88	1
Hypertension	25 (36.8%)	11 (28.9%)	6 (40.0%)	8 (53.3%)	0.25	0.23
Diabetes mellitus	13 (19.1%)	7 (18.4%)	3 (20.0%)	3 (20.0%)	0.99	1
Hypercholesterolemia	33 (48.5%)	19 (50.0%)	6 (40.0%)	8 (53.3%)	0.74	0.90
Current smoking	43 (63.2%)	26 (86.4%)	8 (53.3%)	9 (60.0%)	0.57	1
Body Mass Index, kg/m2	26.1 [24.0, 29.4]	26.0 [23.6, 29.3]	28.5 [26.3, 29.3]	25.8 [23.4, 30.0]	0.45	0.75
Heart rate, bpm	77 ± 17	75 ± 15	80 ± 19	78 ± 20	0.62	0.77
Systolic BP, mmHg	142 ± 25	145 ± 28	134 ± 22	145 ± 22	0.39	0.65
Diastolic BP, mmHg	85 ± 19	87 ± 16	78 ± 21	86 ± 22	0.26	0.83
Anterior STEMI	27 (39.7%)	15 (39.5%)	6 (40.0%)	6 (40.0%)	1	1
Symptoms to balloon, hours	4.7 [3.1, 8.7]	4.5 [3.2, 6.0]	3.7 [2.7, 8.6]	6.8 [3.9, 10.8]	0.36	0.18
Thrombolysis	2 (2.9%)	1 (2.6%)	0	1 (6.7%)	0.69	0.40
GFR, mL/min/1.73m^2^	93 ± 17	91 ± 16	96 ± 19	93 ± 16	0.65	0.88

*BP* blood pressure, *GFR* glomerular filtration rate, *STEMI* ST-elevation myocardial infarction

**Table 2:** Biological and echocardiographic data according to neprilysin levels. ***p < 0.001 vs. baseline

	Overall (*n* = 68)	Neprilysin level ≤ 125 pg/mL (*n* = 38)	Neprilysin level 126–450 pg/mL (*n* = 15)	Neprilysin level > 450 pg/mL (*n* = 15)	Overall *p*.value	Highest-level vs. lower groups *p*.value
At baseline						
NT-proBNP, pg/mL	187 [64, 790]	184 [66, 1155]	287 [87, 566]	217 [65, 1103]	0.77	0.47
hs-cTnI, pg/mL	1947 [159, 9948]	1511 [156, 9794]	1136 [327, 6711]	2765 [574, 10,492]	0.64	0.38
Neprilysin, pg/mL	125 [125, 432]	–	350 [183, 411]	1070 [781, 1824]		
At baseline						
EDV, mL/m^2^	53.8 ± 13.0	55.1 ± 12.4	56.0 ± 13.5	48.3 ± 13.3	0.18	0.07
ESV, mL/m^2^	28.2 [22.4, 34.7]	27.8 [23.2, 34.9]	28.8 [24.3, 32.8]	28.5 [21.1, 36.0]	0.97	0.82
LVEF, %	45.0 ± 8.5	46.4 ± 8.3	47.1 ± 8.1	39.1 ± 6.9	< 0.01	< 0.01
At follow-up						
EDV, mL/m^2^	56.6 ± 15.0	57.8 ± 13.6	58.7 ± 19.9	51.6 ± 12.6	0.35	0.15
%increase in EDV	4.6 [−8.6, 18.6]	−0.1 [8.9, 17.4]	5.9 [−11.4, 22.1]	10.7 [−5.3, 20.5]	0.69	0.39
ΔEDV, mL/m^2^	5.4 ± 23.2	5.5 ± 22.7	5.4 ± 28.6	5.1 ± 20.0	1	0.96
ESV, mL/m^2^	25.4 [19.0, 34.8]	25.8 [19.6, 32.7]	25.9 [18.9, 36.5]	25.1 [19.1, 32.3]	0.91	0.66
%increase in ESV	−14.3 [−23.3, 10.5]	−10.9 [−22.1, 9.7]	−11.1 [−27.0, 28.8]	−15.2 [−24.2, 1.6]	0.69	0.44
ΔESV, mL/m^2^	−4.2 ± 17.7	−3.7 ± 17.3	−0.8 ± 20.9	−8.9 ± 15.4	0.46	0.26
LVEF, %	51.9 ± 9.2***	53.0 ± 8.9***	50.6 ± 9.7	50.4 ± 9.9***	0.55	0.49
%increase in LVEF	17.7 ± 22.0	16.0 ± 19.4	9.1 ± 21.5	30.3 ± 24.5	0.022	0.01
ΔLVEF, %	7.0 ± 8.3	6.6 ± 7.3	3.6 ± 9.0	11.3 ± 8.4	0.031	0.02

*EDV* end-diastolic volume, *ESV* end-systolic volume, *hs-cTnI* high-sensibility cardiac troponin I, *LVEF* left ventricular ejection fraction

**Table 3:** Drug therapy according to neprilysin levels, at baseline and at follow-up

	Overall (*n* = 68)	Neprilysin level ≤ 125 pg/mL (*n* = 38)	Neprilysin level 126–450 pg/mL (*n* = 15)	Neprilysin level > 450 pg/mL (*n* = 15)	Overall *p*.value	Highest-level vs. lower groups *p*.value
At baseline						
antiplatelet agent	6 (8.8%)	2 (5.3%)	1 (6.7%)	3 (20.0%)	0.23	0.23
beta-blocker	3 (4.4%)	2 (5.3%)	0	1 (6.7%)	0.63	1
ACEI/ARB/MRA	18 (26.5%)	8 (21.1%)	5 (33.3%)	5 (33.3%)	0.53	0.73
statins	17 (25.0%)	9 (23.7%)	4 (26.7%)	4 (26.7%)	0.97	1
diuretics	7 (10.3%)	3 (7.9%)	2 (13.3%)	2 (13.3%)	0.77	1
At follow-up						
antiplatelet agent	68 (100.0%)	38 (100.0%)	15 (100.0%)	15 (100.0)%	–	
beta-blocker	64 (94.1%)	36 (94.7%)	14 (93.3%)	14 (93.3%)	0.98	1
ACEI/ARB/MRA	52 (76.5%)	30 (78.9%)	12 (80.0%)	10 (66.7%)	0.60	0.51
statins	65 (95.6%)	37 (97.4%)	15 (100.0%)	13 (86.7%)	0.15	0.24
diuretics	11 (16.2%)	7 (18.4%)	2 (13.3%)	2 (13.3%)	0.86	1

**Fig. 2:**
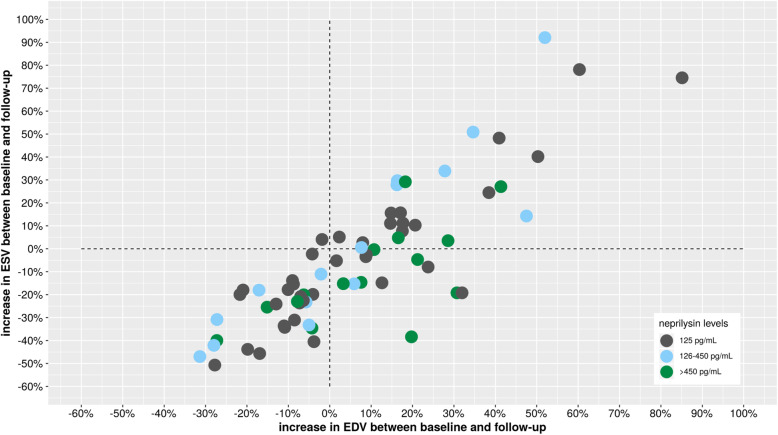
Relationship between plasma neprilysin level at admission and changes regarding left ventricular volumes between baseline and follow-up

**Fig. 3:**
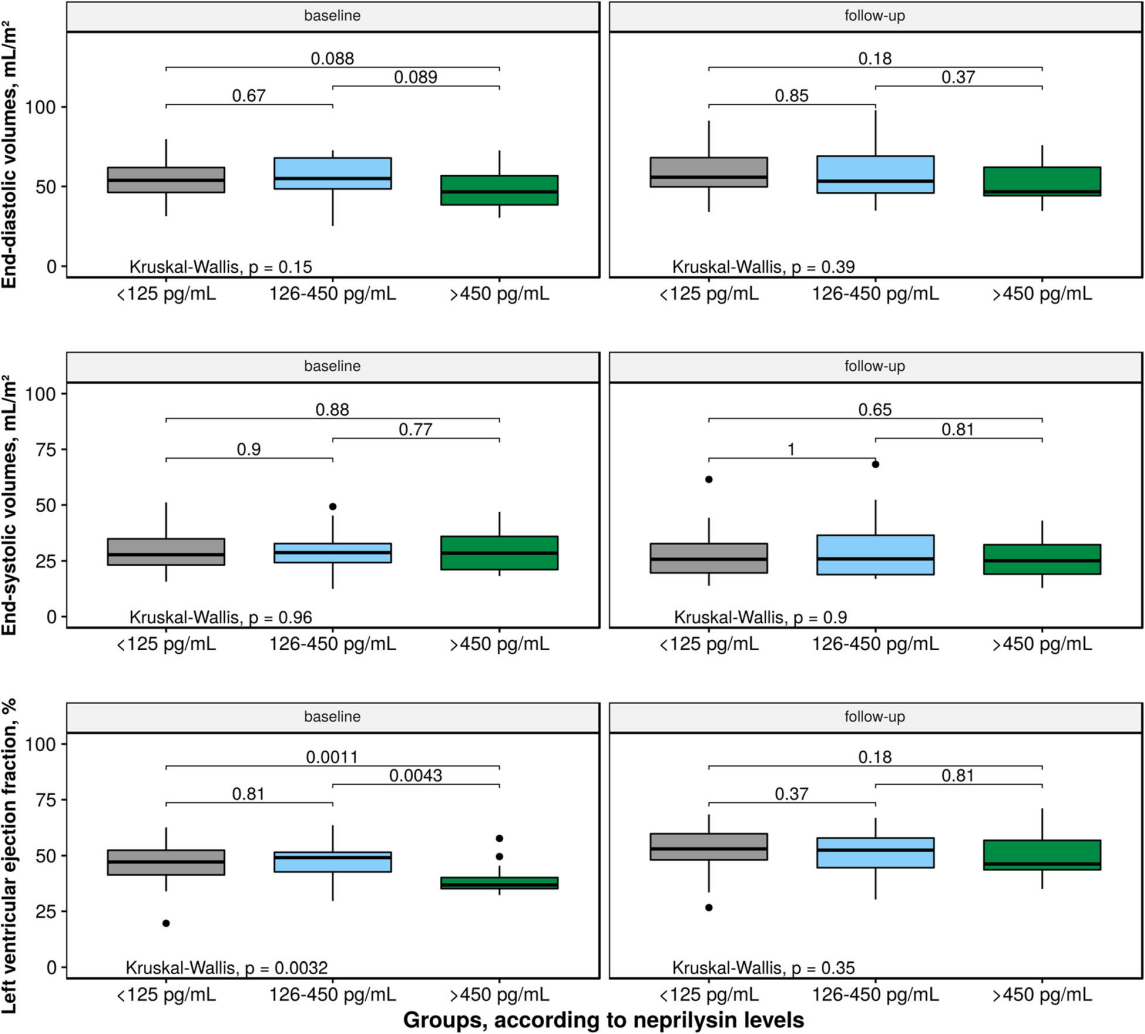
Left ventricular volumes and ejection fraction at baseline and during follow-up

**Fig. 4:**
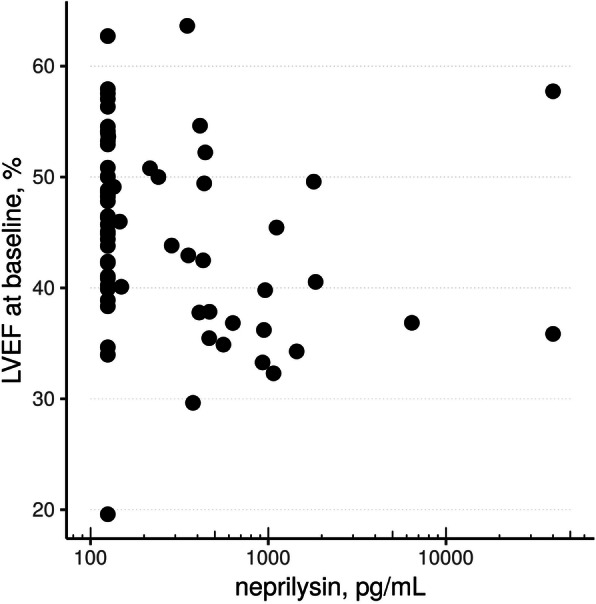
Correlation between neprilysin levels and LVEF at baseline

As shown in Table 1, baseline characteristics were comparable between groups (low-level of neprilysin group [≤125 pg/mL, *n* = 38], medium-level of neprilysin group [126–450 pg/mL, *n* = 15] and a high-level group [> 450 pg/mL, n = 15]). Similarly, as shown in Table 2, baseline NT-proBNP levels and hs-cTnI levels were similarly distributed between groups with no correlation between the levels of the two latter and neprilysin levels (r = 0.06 and r = 0.08, respectively). The relationship between neprilysin levels and the changes of LV volumes between baseline and follow-up is depicted in Fig. 2. There were no significant correlation between levels of NT-proBNP, hs-cTnI or neprilysin and the EDV change between baseline and follow-up (r = 0.07, r = 0.09 and r = 0.07, respectively). At baseline there was a non-significant trend towards lower EDV but significantly lower LVEF in the high neprilysin group (*p* = 0.07 and *p* < 0.01, respectively, vs. other groups, Table 2 and Figs. 3 and 4). During follow-up, LVEF increased in both low and the high neprilysin level groups (46.4 ± 8.3% to 53.0 ± 8.9%, *p* < 0.001; and 39.1 ± 6.9% to 50.4 ± 9.9%, *p* < 0.001 respectively). The magnitude of LVEF increase during follow-up was significantly more important in the high neprilysin group compared to the other groups (*p* = 0.022 for relative and *p* = 0.031 for absolute change in LVEF, Table 2) resulting in similar LVEF levels at follow-up between all groups. Neprilysin values were independently associated with an improvement of LVEF during follow-up in multivariate analysis (Table 4). There was no ischemic event during follow-up. Six patients underwent planned coronary revascularization after the index hospitalization.

**Table 4:** LVEF improvement at follow-up: univariate and multivariate analysis

	Correlation coefficient, [95%CI]	Univariate *p*.value	Multivariate beta coefficient, [95%CI]	Multivariate *p*.value
Age	−0.04 [−0.20, 0.12]	0.63		
Gender, male	0.97 [−4.30, 6.24]	0.71		
Hypertension	− 0.16 [−4.34, 4.01]	0.94		
Diabetes mellitus	2.50 [−2.58, 7.59]	0.33		
Hypercholesterolemia	2.77 [−1.20, 6.74]	0.17		
Current smoking	−3.90 [−7.95, 0.17]	0.06	−3.41 [−7.35, 0.53]	0.09
Body Mass Index,	0.14 [−0.30, 0.58]	0.52		
Heart rate	0.13 [0.02, 0.24]	0.03	0.09 [−0.03, 0.20]	0.13
Systolic BP	0.04 [−0.04, 0.12]	0.30		
Diastolic BP	−0.03 [− 0.14, 0.08]	0.57		
Anterior STEMI	1.79 [−2.30, 5.88]	0.38		
Symptoms to balloon	−0.17 [−0.55, 0.20]	0.36		
GFR	0.06 [−0.07, 0.19]	0.36		
log neprilysin	1.85 [0.31, 3.40]	0.02	1.62 [0.13, 3.11]	0.03
log NT-proBNP	−0.98 [−2.17, 0.22]	0.11	−0.99 [− 2.12, 0.13]	0.08
log hs-cTnI	−0.43 [−1.26, 0.40]	0.30		

## Discussion

The present study is the first to show that after admission for STEMI highest levels of plasma neprilysin are associated with lower LVEF and a trend towards lower EDV at baseline, and higher magnitude of improvement of LVEF at follow-up when compared to other groups. These findings suggest that high neprilysin levels may identify patients with stunned myocardium early after STEMI, with a recovery of contractility leading to improved LVEF at follow-up.

Neprilysin is a neutral endopeptidase that degrades several endogenous vasoactive peptides, such as natriuretic peptides, Angiotensin-II, Endothelin-1, bradykinin, substance P and adrenomedullin [9, 10] which may be involved in the post STEMI neurohormonal activation. More than half of the patients (55.9%) had a very low levels of neprilysin at baseline, below the measurement threshold of 125 pg/mL in the present study. Serial measurements of serum neprilysin concentration following STEMI in a prior study have shown comparable results to our study with a median initial neprilysin level of 88.3 pg/mL [IQR: 14, 375.5]) [14]. Low levels of neprilysin have been reported to be associated with cardiovasciular risk factors such as hypertension and smoking as well as diastolic left ventricular dysfunction in a large community-based cohort of 1536 participants without known cardiovascular disease (median of 3.9 ng/mL [IQR: 1.0, 43.0 ng/mL] [15]). The latter two studies failed to show an impact of neprilysin levels on outcome. These data support a complex relationship between neprilysin levels and cardiac functional or structural compromise in possibly different directions in different conditions. Such complex relationship is not paradoxical as neprilysin degrades effectors with opposite effects. Its action, up- or down-regulation, and maybe inhibition may therefore lead potentially to either beneficial or detrimental effects.

After STEMI, early neurohormonal activation occurs in order to maintain hemodynamic homeostasis [16]. Our study was limited to patients without HF or severely reduced LVEF. We found a mild increase in natriuretic peptides levels and mildy reduced LVEF in the study population. Neprilysin catalyzes the degradation of several vasodilator peptides, especially natriuretic peptides but also bradykinin, substance P, and adrenomedullin [17]. It is likely that the increase of the level of these peptides is at least partly related to a decrease of plasma neprilysin concentrations although the direction of such relationship could not be identified. In the above mentioned large community-based cohort, excluding patients with a LVEF< 45%, low neprilysin levels were associated with higher prevalence of smokers, hypertension, dyslipidemia and impaired diastolic function [15]). Although the level of plasma neprilysin were not available before the onset of STEMI in our study, it may be speculated that most patients included in our analysis, with cardiovascular risk factors, may have had low levels of neprilysin prior to acute myocardial infarction.

The associations between low soluble neprilysin levels and an adverse cardiometabolic and smoking profile in the general population on one hand and the association between high levels and poor outcome [18–20] as well as the beneficial effect of simultaneous neprylisin and angiotensinII inhibition in HF with altered LVEF populations [12] may be explained by different populations and different levels and patterns of neurohormonal activation. Endothelial dysfunction and the subsequent vasoconstriction, are common findings in patients with coronary artery disease risk factors (hypertension, smoking, dyslipidemia) [21]. Neprilysin degrades atrial natriuretic peptide which is a key signaling pathway in blood pressure regulation [22]. The association between such risk factors and the lower levels of neprilysin in the general population may be explained by the down-regulation of neprilysin in this setting to counteract impaired vasomotion, while in the setting of HF, high levels of neprilysin, upregulated by the increase of natriuretic peptides levels, are associated with detrimental effects which may be prevented by its inhibition [12].

The present STEMI population is different from both the general population and the chronic or acute HF patient populations. Hemodynamic status and the evolution of cardiac volumes and function are obviously different between patients without cardiovascular disease, with subclinical diastolic LV dysfunction, HF with preserved or decresed LVEF and finally the acute ischemic injury and LV overload of STEMI. The impact of neurohormonal activation after STEMI on cardiac remodeling increases as the reparative and proliferative phases begin. The post STEMI left ventricular overload leads to increased proBNP production by left ventricular cardiomyocytes [23]. Neprilysin clears BNP from circulation, resulting in a limitation of its adaptative natriuretic action [22]. There are controversial data about the ability of natriuretic peptides levels at admission to predict LVR following STEMI [24–26]. Our study, in concordance with prior studies, showed that soluble neprilysin levels were not correlated to natriuretic peptide levels [15] at the time of measurement, early after STEMI. Accordingly, we observed no correlation between initial assessment of neprilysin levels and adverse remodeling during follow-up. These results, in concordance with a prior study [14] suggest that neprilysin may not be an early biomarker of adverse remodeling after STEMI. Unlike the prior study, we assessed dynamic changes of LV volumes and function. The lower LVEF observed in STEMI patients with the highest levels of neprilysin in our study was driven by a non-significant lower EDV at baseline. Interestingly, these patients showed significant improvement of LVEF at follow-up when compared to other groups. These findings arise the hypothesis of a relationship between high levels of neprilysin and the extent of stunned myocardium, with low contractility at baseline and recovery at follow-up following successful myocardial reperfusion. One speculative explanation to our findings may be that in patients with extended stunned myocardium, levels of BNP may be high very early after STEMI, leading to an up-regulation of neprilysin, leading itself to a reduction of BNP levels at the time of measurement. In such patients without LV enlargement and heart failure higher levels of neprilysin may only be a marker of stunned myocardium, recovering at follow-up. Other hypotheses with a beneficial action of high levels of neprilysin, as a direct or indirect consequence of post-STEMI neurohormonal activation, acting more effectively on vasoconstrictive/pro-proliferative peptides may be considered. Bernelin et al. showed a non-significant decrease of neprilysin levels following STEMI, from baseline, to 1 month [14]. This non-significant trend (*p* = 0.70) may possibly be related to the small sample size (*n* = 21) in the latter study. The latter study evaluated LV volumes and function only once at 1 month, hence not allowing the analysis of changes in such parameters. In our study neprilysin level measurements were performed only once, on admission, hence not allowing any analysis of the impact of neprilysin level kinetics on the studied parameters. It may be interesting to assess the evolution of neprilysin concentrations and the evolution of cardiac imaging parameters in a larger STEMI population at different timpoints.

The present study has several limitations. The analysis includes a small number of patients. The population is highly selected including only patients with successful myocardial reperfusion, without heart failure and who had complete 3D-transthoracic echocardiogram at 2 timepoints and a collection of blood samples. Because of a high drop-out rate a selection bias could not be excluded. Our results could not be generalized to all settings of STEMI. Neprilysin has complex pleiotropic effects and is involved in different pathways and our study may not assess the pathophysiological bases of our findings. Moreover, the absence of correlation between circulating neprilysin concentration and activity has been previously reported [27].

## Conclusions

Our study shows that after admission for STEMI, in a selected population with successful myocardial reperfusion and no clinical sign of HF, highest levels of plasma neprilysin are associated with lower LVEF at baseline, a trend towards lower EDV and higher magnitude of improvement of LVEF at follow-up when compared to other groups. These findings suggest that high neprilysin levels may identify patients with stunned myocardium early after STEMI, with a recovery of contractility leading to improved LVEF at follow-up. If confirmed by other large sized studies, neprilysin level measurements may contribute to identifying patients with decreased LVEF at baseline but who are more likely to recover at follow-up. Further studies are also warranted to assess the impact of neprilysin in the general setting of STEMI and its potential blockade.

## Data Availability

The datasets used and/or analysed during the current study are available from the corresponding author on reasonable request.

## Abbreviations

ACE: Angiotensin-converting enzyme
AT1: Angiotensin-II receptor type 1
BNP: Brain natriuretic peptide
EDV: End-diastolic volume
ESV: End-systolic volume
HF: Heart failure
hs-cTnI: High-sensitivity cardiac troponin I
LV: Left ventricular
LVEF: Left ventricular ejection fraction
LVR: Left ventricular remodeling
NTpro-BNP: N-terminal pro-B-type natriuretic peptide
pPCI: Primary percutaneous coronary intervention
RAAS: Renin-angiotensin-aldosterone system
STEMI: ST-elevation myocardial infarction
